# Hyperspectral Imaging and K-Means Classification for Histologic Evaluation of Ductal Carcinoma *In Situ*

**DOI:** 10.3389/fonc.2018.00017

**Published:** 2018-02-07

**Authors:** Yasser Khouj, Jeremy Dawson, James Coad, Linda Vona-Davis

**Affiliations:** ^1^Lane Department of Computer Science and Electrical Engineering, West Virginia University, Morgantown, WV, United States; ^2^Department of Pathology, West Virginia University, Morgantown, WV, United States; ^3^West Virginia University Cancer Institute, Morgantown, WV, United States; ^4^Department of Surgery, West Virginia University, Morgantown, WV, United States

**Keywords:** hyperspectral, breast cancer, ductal carcinoma, spectral reflectance, hematoxylin and eosin, unstained, K-means

## Abstract

Hyperspectral imaging (HSI) is a non-invasive optical imaging modality that shows the potential to aid pathologists in breast cancer diagnoses cases. In this study, breast cancer tissues from different patients were imaged by a hyperspectral system to detect spectral differences between normal and breast cancer tissues. Tissue samples mounted on slides were identified from 10 different patients. Samples from each patient included both normal and ductal carcinoma tissue, both stained with hematoxylin and eosin stain and unstained. Slides were imaged using a snapshot HSI system, and the spectral reflectance differences were evaluated. Analysis of the spectral reflectance values indicated that wavelengths near 550 nm showed the best differentiation between tissue types. This information was used to train image processing algorithms using supervised and unsupervised data. The K-means method was applied to the hyperspectral data cubes, and successfully detected spectral tissue differences with sensitivity of 85.45%, and specificity of 94.64% with true negative rate of 95.8%, and false positive rate of 4.2%. These results were verified by ground-truth marking of the tissue samples by a pathologist. In the hyperspectral image analysis, the image processing algorithm, K-means, shows the greatest potential for building a semi-automated system that could identify and sort between normal and ductal carcinoma *in situ* tissues.

## Introduction

Breast cancer is one of the highest causes of cancer deaths among American women (1–4). According to the U.S. Breast Cancer Organization, statistics show that about one in eight U.S. women will develop invasive breast cancer over their lifetime (1–3). In 2016, about 246,660 new cases of invasive breast cancer were diagnosed in women. The role of the pathologist is undeniably important for cancer diagnosis (4, 5). However, as the number of breast cancer cases increases, the burden of pathological cases becomes onerous. Thus, any new technology that can expedite breast cancer detection and diagnosis using biopsy slides, making the process easier and more efficient, is warranted.

Hyperspectral imaging (HSI) is a sophisticated non-invasive optical imaging modality that has the potential to accelerate medical imaging research and clinical practice. It is an optical imaging modality that collects and analyzes spectral information from across the electromagnetic spectrum, typically spanning the visible wavelengths between 450 and 700 nm, but also extending to the infrared (>700 nm) and UV (<450 nm). HSI has advantages over conventional imaging in that it provides the spectral reflection or absorption characteristics of the object being imaged in the form of spectral channels contained in an image data hypercube (6–8). Preliminary research performed by the WVU Optical Imaging group at the WVU Cancer Institute has demonstrated that HSI and classification methods could distinguish between tumor and normal tissue in animal experiments with different tumor sizes without the use of contrast agents (9). The same researchers imaged pathological slides using a hyperspectral camera and reported the detection of head and neck metastasis *ex vivo* with promising sensitivity and specificity.

The purpose of this study was to evaluate the performance of a snapshot hyperspectral imager, the Arrow system from Rebellion Photonics, to determine if measurable differences in spectral properties exist between normal and various stages of cancerous breast tissues fixed on biopsy slides (10, 11). We hypothesized that a hyperspectral imager could spectrally determine the difference between normal and cancerous tissue on both stained and unstained slides. In tissues verified by a pathologist, we further predicted that image processing techniques could successfully differentiate between tissues in a semi-automated fashion (9, 12–14).

## Materials and Methods

### Instrumentation

The hyperspectral camera was connected to an upright microscope (Nikon Optiphot-2) with at 12V-100W LL halogen lamp illumination source to capture images from biopsy slides. All images were captured using a CF Achromatic P40x objective. The arrow snapshot HSI camera is capable of two imaging configurations with two different sets of spectral bands. The configuration that was used in this study is 443 × 313 pixel resolution in the spatial domain, with 31 bands in spectral domain. The spectral range of this system is within the visible light spectrum between 461 and 641 nm. The field of view of the hyperspectral camera attached to the microscope with a 40× objective in its best resolution is 100 µm × 80 µm, allowing the capture of many images from a single tissue sample, which averaged 8.0 × 10^−5^ cm^2^ for this study.

**Figure 1:**
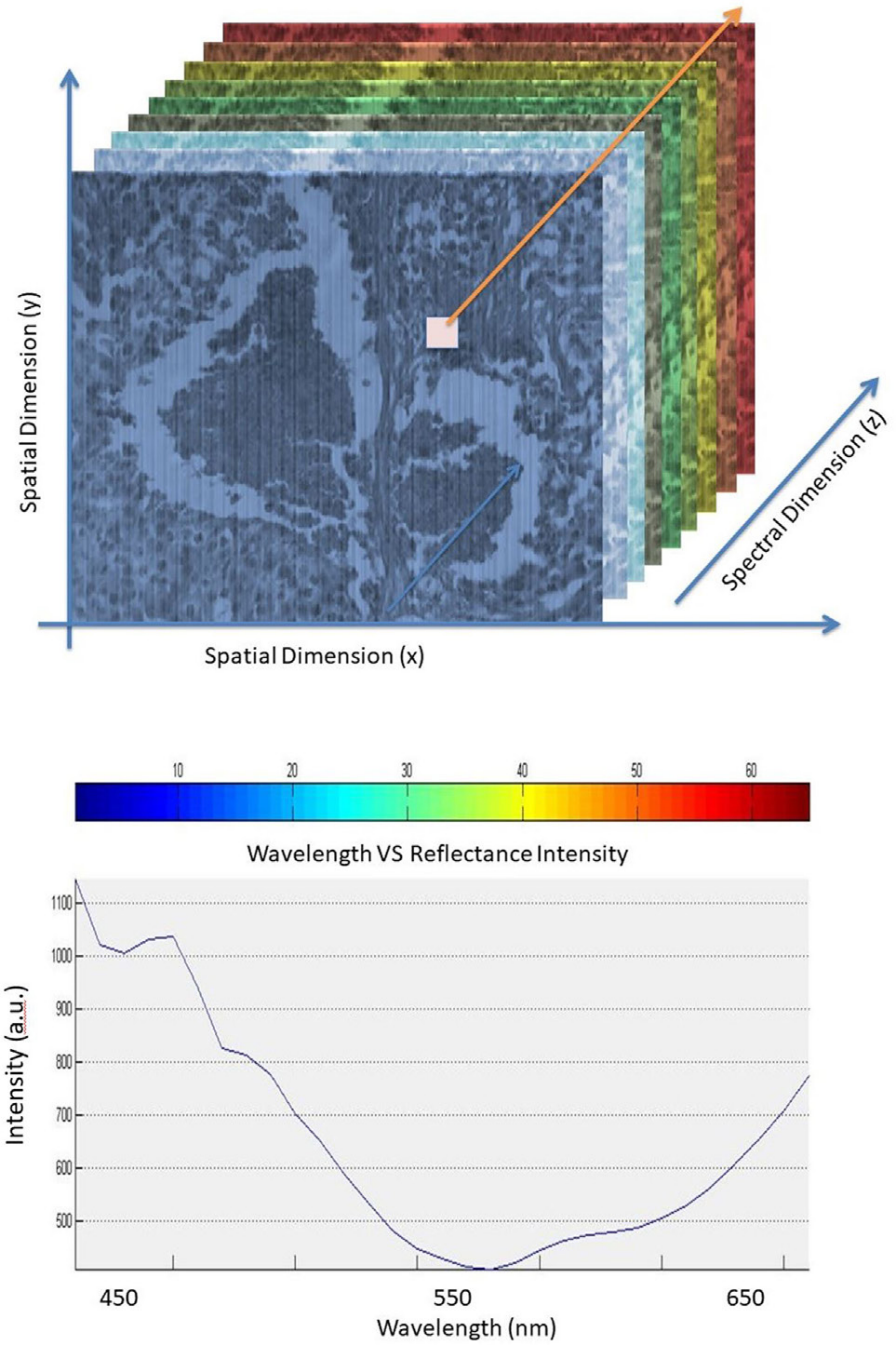
Hyperspectral cube (*top*). Pixel spectrum in the spectral dimension (*bottom*).

An illustration showing the hyperspectral cube and graph of pixel spectrum are shown in Figure 1. The hypercube is essentially a three-dimensional dataset, which means even a single pixel contains a feature vector with over 10 dimensions within the entire spectrum of reflectance information (15, 16). The technology of the hyperspectral imager requires using optical sensors, which are adjusted to collect spectral information in several narrow bands or channels. Typically, these channels range between 2 and 10 nm wide. HSI sensors produce image data in dozens or hundreds of narrow spectral bands (17–21). HSI technology, when properly exploited, can be used in optical imaging applications such as optical medical imaging in clinical and research applications such as the work described here focused on breast cancer tissue detection (22, 23).

**Figure 2:**
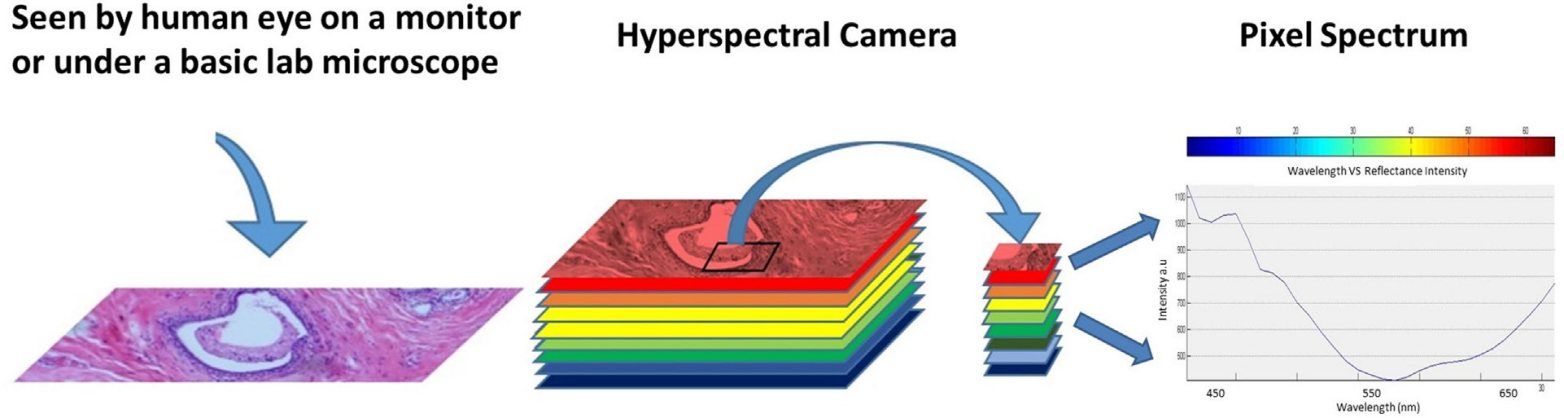
Regular image of the breast tissue, hyperspectral image, and the spectrum of the marked pixel.

The information collected from the imaging system was analyzed to obtain a continuous spectrum for each image pixel or cell as shown in Figure 2. Areas targeted by the imaging system may have different biological, and chemical compositions that can be detected by the sensor due to differences in light reflection or absorption. The image spectra can be compared with reflectance spectra collected in the field or in a laboratory to recognize and map surface materials such as particular types of vegetation or diagnostic minerals associated with ore deposits (20). Hyperspectral images contain a rich collection of data, which requires an understanding of the optical properties of different materials, how they are being measured, and how they relate to the measurements made by the hyperspectral sensor (20).

There are several methods used to create hyperspectral imagery. When comparing between different methods, such as hyperspectral line scan and hyperspectral snapshot imaging, it is important to consider both imaging techniques, as well as advantages and disadvantages of both systems (24, 25). In the case of line scan, each image collects one wavelength after the other, and the field of view of the imaging system is fixed to build the hypercube (26, 27). However, in the case of the snapshot method, both spatial and spectral information of the target are captured with one exposure (28).

**Figure 3:**
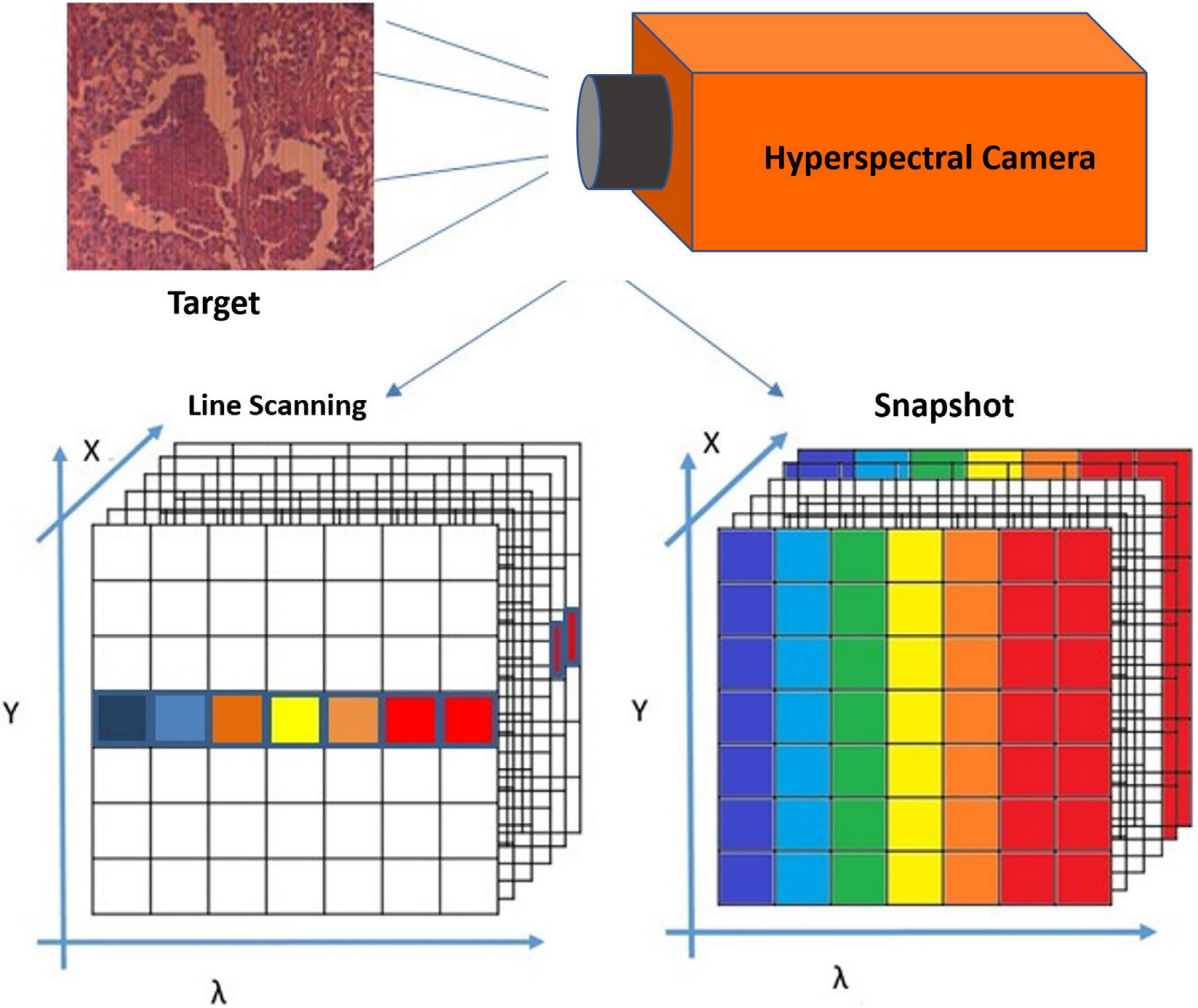
Building the spectral data cube in both line scan and snapshot systems.

The snapshot method (used in this study) is an imaging technique that does not require scanning at all. As shown in Figure 3, the snapshot camera has the capability to produce a complete spectral data cube in a single integration by directly imaging the target zones onto the spectral, and spatial detectors simultaneously (28–30). In the line scan method, time and stability are required, and it is necessary to wait until all wavelength images have been recorded, which takes between several seconds to minutes of measurement time, in addition to a few seconds wait time between scans, depending on the imaged target and the imager capability from illumination and integration conditions (31).

### HSI Image Processing and Data Analysis

Image processing consists of a mathematical algorithm that, when applied, derives information from an image that can be used in automated image analysis systems. K-means is a learning algorithm used to solve unsupervised data classification problems (15, 32). Unsupervised clustering is used to describe processes where a classifier is assigned a dataset without preexisting labels (33, 34). For hyperspectral data, the classifier can be used to find spectral classes in a multiband image without assigned values from the data provider. The clustering treats unsupervised data by providing access to the tools to learn the classification from the data itself to create clusters that group the data based on the desired classification (34).

The idea is to define k centers, one for each cluster. Ideally, the best choice is to place them as far away from each other as possible, to increase the detection accuracy, and to reduce the number of error events (35). The next step is to take each point belonging to a given data set and associate it to the nearest center. In this case, each class will have one centroid, and the result becomes more stable with increased iterations, which means they reach a consensus. Also, the more data the classifier is given, the better the centroid accuracy will be (35, 36).

Equations 1 and 2 describe the basic process of choosing clusters in the K-means algorithm. First, this algorithm minimizes the objective function, *J(V)*, is calculated using [Ref. (36)]:

(1) $$J(V)=\sum_{i=1}^{C}\sum_{i=1}^{C}{\left(∥\mathrm{xi}-\mathrm{vi}∥\right)}^{2}$$

where “||*xi* − *vj*||” is the Euclidean distance between *xi* and *vj*. “*ci*” is the number of data points in *i*th cluster, and “*c*” is the number of cluster centers.

The process starts by randomly selecting “*c*” cluster centers, then calculating the distance between each data point and cluster centers. After that, a data point is assigned to the cluster center whose distance from the cluster center is the minimum of all the cluster centers.

The new cluster center, *vi*, is recalculated using the minimum-distance classifier equation [Ref. (35)]:

(2) $$\mathrm{vi}=\left(\frac{1}{\mathrm{ci}}\right)\sum_{j=1}^{\mathrm{ci}}\mathrm{xi}.$$

Then, the distance between each data point and newly obtained cluster centers is recalculated. Finally, if no data point was reassigned, the calculation loop is stopped. Otherwise, it is repeated until no changes in the centroid values occur (35).

### Cellular Imaging

**Figure 4:**
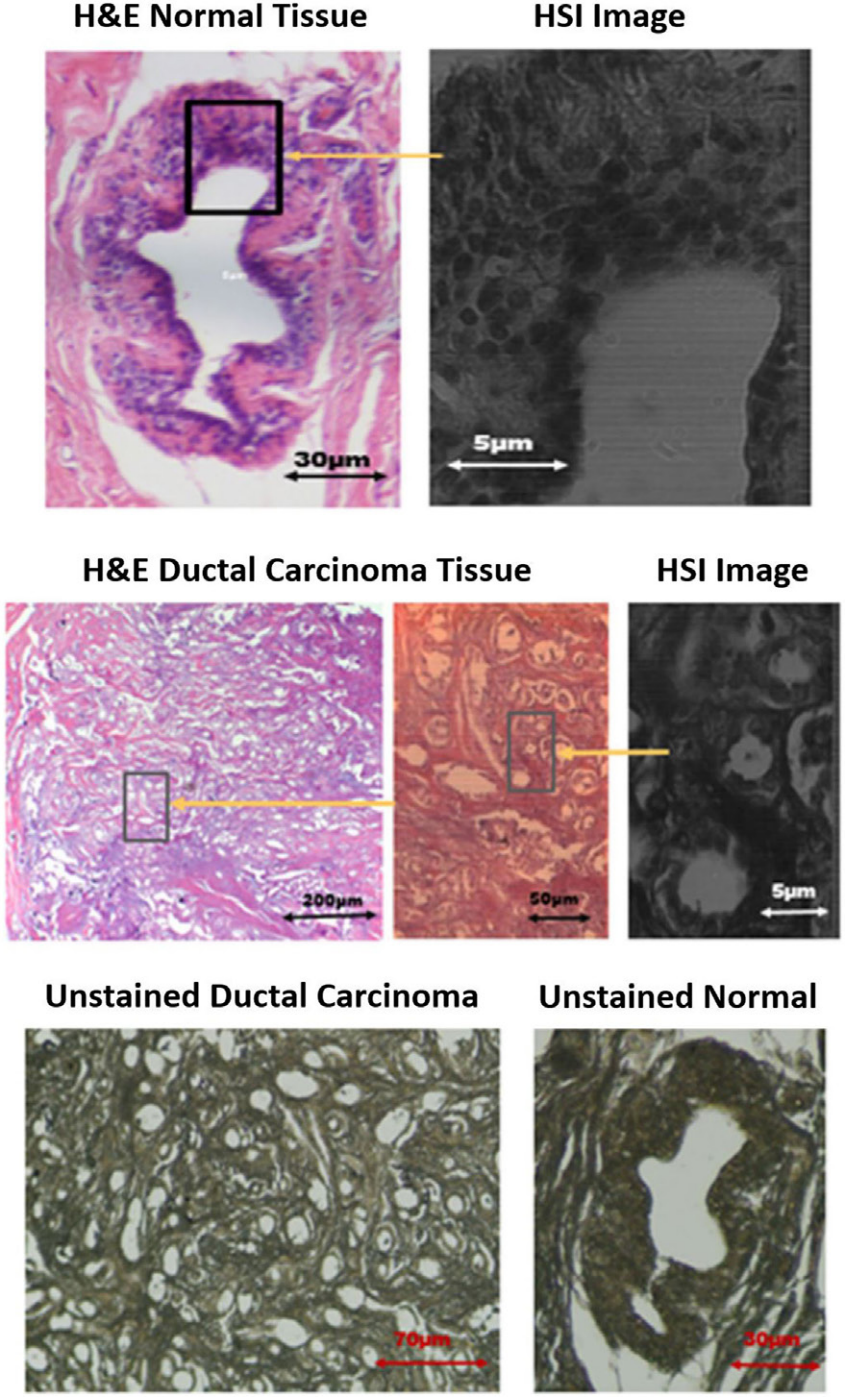
Images of stained and unstained samples. The marked [hyperspectral imaging (HSI)] are representing the chosen wavelength channel (550 nm) for detection.

This section provides a description of the snapshot HSI of human breast cancer tissue [obtained under WVU IRB protocol #1509816662 (Non-Human Subject Research)]. The samples are grouped as follows: (1) hematoxylin and eosin (H&E) stained normal and ductal carcinoma *in situ* (DCIS) samples and (2) unstained samples of normal and DCIS tissues. In the first imaging experiment, the HSI system was applied on 10 samples from patients that have tested positive for invasive ductal carcinoma breast cancer. Each patient has two H&E tissue samples marked by a pathologist. The second imaging experiment includes set of breast tissue samples taken from the same 10 patients, but without staining to observe the spectral signature of both normal and cancer tissues in an unstained state. The thickness of each sample was 5 μm. Example images from biopsy slides shown in Figure 4 for a single subject displaying both normal and DCIS tissue DCIS. Each slide was imaged by the hyperspectral imager. Figure 4 shows the H&E stained and unstained samples of the normal ducts, and DCIS of the patient with a monochromatic hyperspectral image at the chosen wavelength channel. A visible image of H&E-stained as well as -unstained normal duct, and DCIS tissue are shown for the example subject, as well as the regions that were selected for HSI for select wavelengths ranging from 460 to 650 nm.

## Results and Discussion

**Figure 5:**
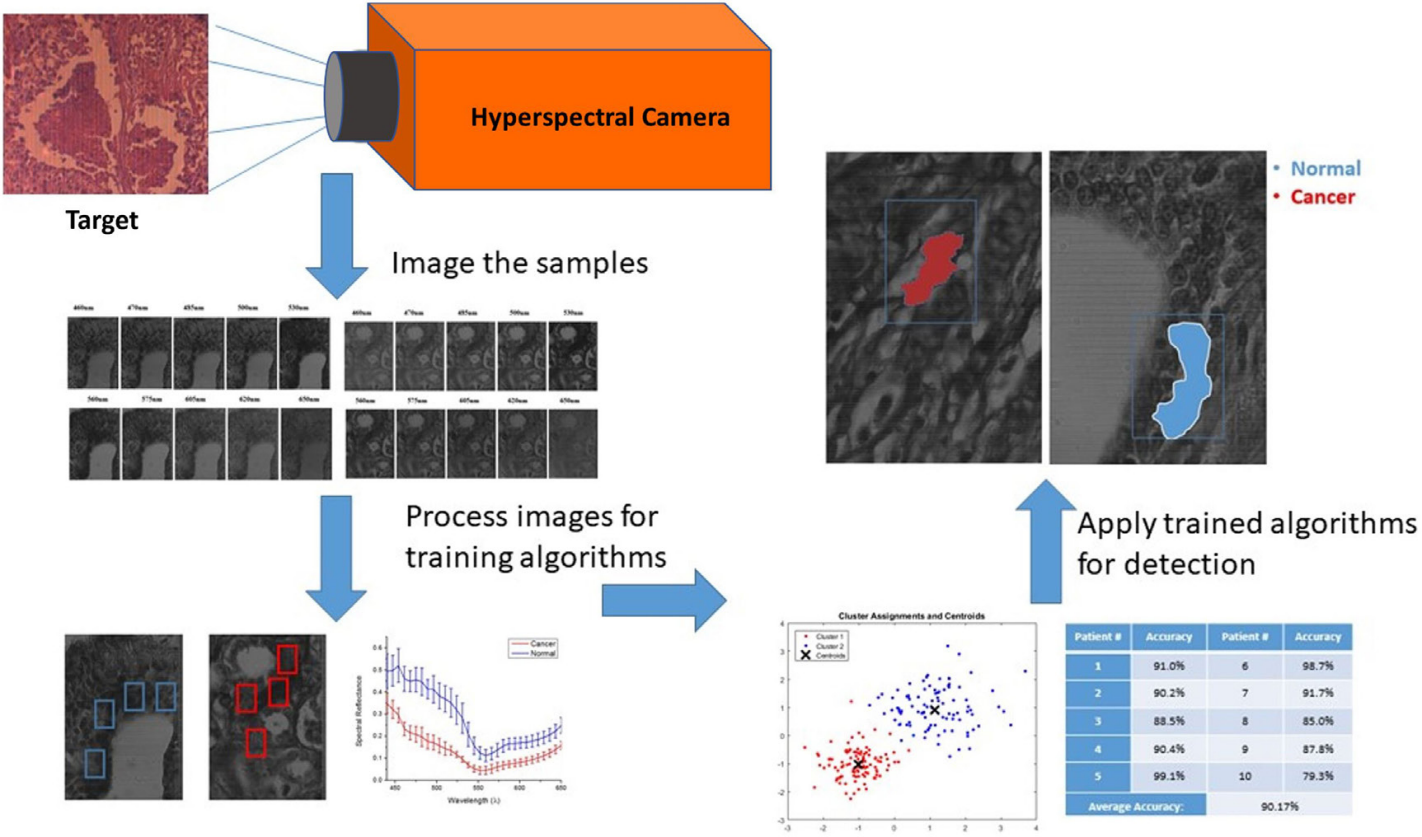
Hyperspectral imaging, training, and semi-auto detection workflow.

The large amount of data contained in the three-dimensional hyperspectral data cube was evaluated in multiple steps as shown in Figure 5. All images were manually marked by a pathologist for providing ground-truth description of the tissue samples showing evidence of cancer. Training the auto detection algorithm and evaluating the accuracy on tissue samples that are not included in the training set is an important step. All marked areas on the samples possessed a high density of tumor tissue within the designated region, as identified by the pathologist. Image processing techniques were applied to look at the regions of interest identified by the pathologist and, ultimately, determine the spectral reflectance of tissues in these regions over the visible light wavelength spectrum. The image processing and HSI toolboxes contained in both MATLAB and Waikato Environment for Knowledge Analysis (WEKA) were used in image processing. WEKA is a collection of machine learning algorithms for data mining tasks that were developed at University of Waikato, New Zealand. In this work, the algorithms are used to learn the about the nature of the hyperspectral data to understand the spectral reflectance value and how it can be used to differentiate between different regions in the tissue samples. We analyzed the manually picked regions of the hyperspectral images, then plotted the spectral reflectance spectrum to compare between the tissues. A semi-automated algorithm was applied to both the supervised, and unsupervised (i.e., labeled, and unlabeled) hyperspectral data sets.

### Spectral Reflectance Determination of Manually Marked Cancer Tissue

**Figure 6:**
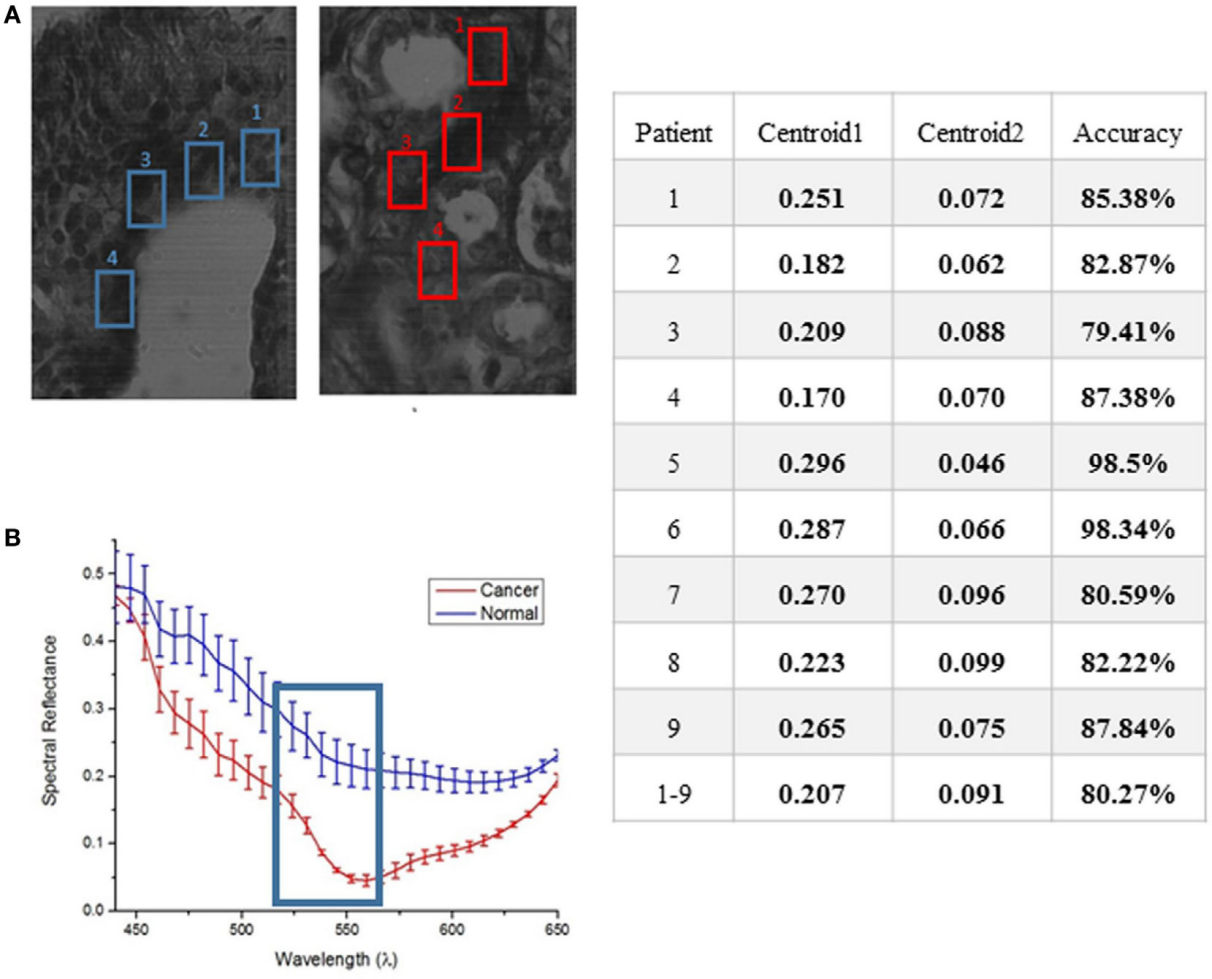
Average response showing spectral reflectance of the cancer and normal tissues **(A)**. The error bars quantify uncertainty in the graph based on an average of 10 measurements taken from each of the four marked region of interest for both normal and cancer tissue **(B)**. A clear separation between both tissues was displayed at 550 nm. The table shows the results of applying K-means separately on each set of data from 9 to 10 subjects for training.

Because ductal carcinoma normally starts spreading from the duct, four square regions were randomly picked around each normal duct and in high density areas of the ductal carcinoma samples known to contain tumorous tissue. The results of hyperspectral images from each patient will consist of a comparison of spectral reflectance measurements from each area, and the average with error and SD computed for 10 measurements from each of 4 separate regions per ample type. Spectral reflectance plots of the 4 marked areas of the stained tissue samples of 1 out of 10 patients are shown in Figure 6.

### Unsupervised Data for K-Means Algorithm on Human—Female H&E-Stained Breast Tissue

The method used to process and analyze images were contained in the HSI toolboxes of MATLAB and WEKA, as described previously. The K-means algorithm was applied to unsupervised (i.e., unlabeled) data. The user chooses the number of classes, which is 2 for this study (cancer, and non-cancer), and then the algorithm picks the best centroid for each class. The class centroids changes each time the algorithm runs until the program stops, i.e., when the numbers of each class do not change, and each centroid is considered the best pick. The table in Figure 6 shows the results of applying K-means separately on each set of data from 9 out of 10 patients for training, iterating to find the two best values of centroids, and finally, using the known labels of cancer and non-cancer to measure the accuracy of each run. The last step is to run the K-means trained algorithm on the 10th patient for testing the cancer detection scheme to determine if the classifier can identify and detect both classes, and then compare the detected results with the ground-truth pathologist diagnosis in order to support the algorithm accuracy.

**Figure 7:**
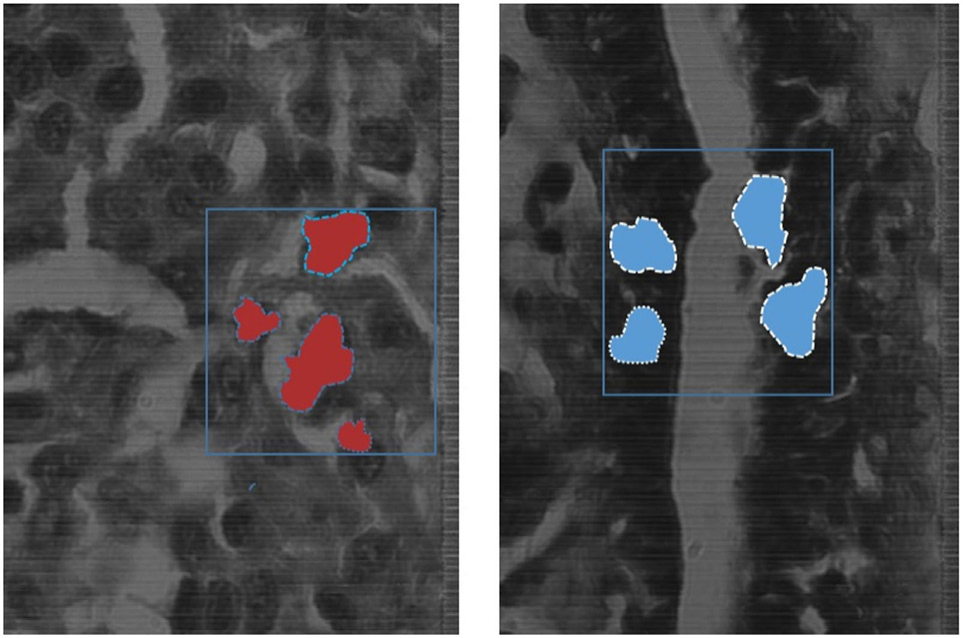
The blue boxes indicates the areas marked by the pathologist, the shaded areas shows the detection of the K-means algorithm detecting cancer tissues (*red*), and normal tissues (*blue*) on the spectral channel 550 nm.

K-means algorithm was applied to the hyperspectral data cubes, and we successfully detected spectral tissue differences with sensitivity of 85.45%, and specificity of 94.64% with a true-negative rate (TNR) of 95.8%, and a false-positive rate (FPR) of 4.2%. The result of training the K-means algorithm on the data that were extracted from the first 9 patients, then the trained algorithm was applied on the untrained data that were extracted from the 10th patient. The algorithm successfully detected normal and cancer tissue as shown in Figure 7. In this figure, the blue boxes indicate the areas containing cancer marked by the pathologist, and the red and blue shaded regions are the regions with cancer identified by the hyperspectral detection algorithm.

## Conclusion

The goal of this study is to evaluate the performance of a snapshot hyperspectral imager to see if measurable differences in spectral properties can be observed between normal and various stages of cancerous breast tissues fixed on biopsy slides, specifically in the case of ductal carcinoma. This interdisciplinary work may also build a bridge between pathology and hyperspectral optical diagnostic imaging to reduce time and workload on the pathologist, with a secondary benefit of leading to more accurate diagnoses.

Hyperspectral imaging technology was used to image stained and unstained tissue samples of normal and DCIS tissue from breast cancer patients. A region of interest in each tissue sample was manually chosen for testing to evaluate the effectiveness of automated cancer detection in these samples. The imaging results showed clear spectral reflectance separation between stained normal and ductal carcinoma tissues, with a TNR of 95.8%, and a FPR of 4.2%. These results indicate that the algorithm could provide a semi-automated “first pass” analysis of histology slides and flag those of interest or concern for further review by a pathologist. In most cases, the results showed the largest separation at the 550-nm wavelength channel of the snapshot hyperspectral camera, making it a good wavelength to choose as starting reference for testing the spectral reflectance of new samples. In the hyperspectral image analysis, the image processing algorithm, K-means, showed the greatest potential of building a semi-automated system that can identify and sort between the samples with a high degree of difference in spectral reflectance. This technique may also be used to distinguish between cancer cells at various stages of progression, as well as to develop a more advanced algorithm to allow a user to distinguish tumor margins from normal and cancerous tissue. Using a HSI system with the development of trained algorithms for detection shows great potential in automating cancer diagnostics in the future.

## Author Contributions

YK, JD, and LV contributed in design, testing, and image analysis highlighted in this article. JC contributed the de-identified biopsy slides highlighted in this article. YK and JD wrote the manuscript highlighting the perspective of using hyperspectral imaging to evaluate ductal carcinoma *in situ*. All authors reviewed the manuscript and provided intellectual contributions.

## Conflict of Interest Statement

All authors declare that the research was conducted in the absence of any commercial or financial relationships that could be construed as a potential conflict of interest.
